# Relationship between vitamin D and asthma from gestational to adulthood period: a meta-analysis of randomized clinical trials

**DOI:** 10.1186/s12890-023-02514-4

**Published:** 2023-06-17

**Authors:** Marharyta Sobczak, Rafal Pawliczak

**Affiliations:** Department of Immunopathology, Division of Biomedical Science, Faculty of Medicine, Medical University of Lodz, Zeligowskiego 7/9 St, 90-752 Lodz, Poland

**Keywords:** Vitamin D, Asthma, Meta-analysis, Randomized clinical trials, Supplementation

## Abstract

**Background:**

Despite numerous studies investigating vitamin D, its impact on asthma is still unknown. The aim of our meta-analysis is to analyze the vitamin D supplementation influence on asthma prevention and treatment ranging from gestational to adulthood period.

**Methods:**

Fifteen randomized clinical trials were included after database search. Studies contained the analyzed endpoints: the number of asthma and wheezing occurrence in gestational and infantile periods, the change of childhood/adult asthma control test score and forced expiratory volume in one second (FEV1) in childhood and adulthood periods. Random effects model was used to calculate effect sizes.

**Results:**

Supplementation by women during pregnancy period decreased the wheezing occurrence in their children by 23% (RR = 0.77; 95% CI [0.64; 0.92]; *p* < 0.0049, I^2^ = 0%); whereas had no effect on given asthma parameters during the infantile period. Moreover, vitamin D administration had negative effect on the FEV1 change in children (MD = -3.84; 95% CI [-7.68; -0.01]; *p* = 0.0497; I^2^ = 95%), but had positive effect on the change of ACT score in adults (MD = 1.80; 95% CI [0.12; 3.49]; *p* = 0.0359; I^2^ = 99%).

**Conclusions:**

Our meta-analysis showed the varying results depending on patient's life period. It is important to further investigate the role of vitamin D supplementation in asthma management.

**Supplementary Information:**

The online version contains supplementary material available at 10.1186/s12890-023-02514-4.

## Introduction

Vitamin D is not only a regulator of calcium and phosphate metabolism, but also acts as an immunomodulator. Humans can get the vitamin D from three major sources: sunlight, food supplements and diet [1, 2]. Vitamin D was discovered in 1922. In 1928 Adolf Windaus received the Nobel Prize for research on the composition of sterols and their relationship with vitamins. However, it was not until 1941 that the vitamin was added to the list of recommended dietary supplements [3]. Nowadays, vitamin D deficiency is a common problem among both children and adults. The proper level of vitamin D is important at any age: starting at pregnancy, where its deficiency may necessitate a caesarean section during childbirth, or cause the development of caries or wheezing in newborn babies. Studies indicate that even in the United States, where vitamin D is added to some dietary products, its levels below 30 ng/ml have been detected in 50% of children under the age of 5 and 70% of children aged 6 to 11 years. This phenomenon may be caused, among others, by an increased incidence of obesity or the excessive use of sunscreen creams [4]. The recommended daily vitamin D intake reaches 400 IU for infants up to 1 year of age; while in children/adolescents from 1 to 18 years – 600 IU. Of note, supplementation is crucial for infants, as even 8 weeks-old infant may develop vitamin D deficiency. Similarly, recommended daily intake of vitamin D differs among adult and elderly groups. Recommended dose of vitamin D for adults under 70 years-old equals 600 IU per day; while past 71 years, 800 IU daily dosage is proposed [5].

Respiratory tract infections may play an important, but controversial role in the etiology of asthma. According to some reports, during early childhood, the infections can cause wheezing or in some cases even protect against the development of asthma and other allergic diseases [6–8]. There are several lines of defense for the airways exposed to potential pathogens: the first one, mucus layer, that covers the ciliated epithelium and contains mucins; while the other one includes the antimicrobial peptides and antimicrobial proteins found in the surface fluid of the airways. It has been proven that low vitamin D levels may increase the risk of respiratory infections and asthma [9]. Unlike numerous in vitro and in vivo studies that indicate that vitamin D may alleviate symptoms of asthma, clinical trials show conflicting results [10]. As mentioned above, there is plenty of studies describing anti-inflammatory and potentially anti-asthmatic role of vitamin D. Considering that asthma may differently affect subjects belonging to a particular age groups, in order to picture potential role of vitamin D in asthma prevention and therapy, there is a vast need to understand its impact on varying life periods.

Currently, most of the studies focus on given periods of patient’s life, therefore fail to describe the model of asthma as a disease that may affect the whole lifespan of the subject. Considering possible positive clinical outcomes of vitamin D supplementation in asthmatic patients, we decided to carry out the meta-analysis of randomized clinical trials (RCTs) that studied the influence of vitamin D supplementation on asthma prevention and treatment ranging from gestational to adulthood period.

## Methods

### Search strategy

This meta-analysis was conducted according to the Preferred Reporting Items for Systematic Reviews and Meta-Analyses (PRISMA) guidelines [11]. The databases, such as PubMed, Embase and the Cochrane Central Register of Controlled Trials, were searched to find literature published as of October 30, 2022. The following keywords were used: *“Vitamin D”, “Vitamin D3”, “25-hydroxyvitamin D”, “Cholecalciferol”, “25(OH)D”, “asthma”, “supplement”, “supplementation”.*

### Study selection and data extraction

Inclusion criteria comprised only articles of blinded control-compared RCTs investigating the topic of vitamin D supplementation in asthma prevention and treatment. Exclusion criteria were as follows: articles not written in English and not containing endpoints, such as: the number of asthma and wheezing events in gestational and infantile periods, the change of childhood asthma control test (C‐ACT) score and forced expiratory volume in one second (FEV1) in childhood period; and the change of asthma control test (ACT) score and FEV1 in adulthood period. If given study presented the data as a median (Q3-Q1), the value was converted into mean according to method presented by Hozo et al. [12]. If the variables that presented the change in ACT score and FEV1 were missing, we calculated it using values reflecting states before and after treatment. SD (Standard deviation) change before and after treatment was calculated according to Cochrane Handbook for Systematic Reviews of Interventions [13] using the formula:

$$SD=\sqrt{{SD}_{before}^{2}+{SD}_{after}^{2}-(2\times Correlation\times {SD}_{before}\times {SD}_{after})}$$

Correlation coefficient was calculated using the study by Jat et al. [14]. In infantile period, study by Rosendahl et al. [15] included two experimental groups with varying doses of the vitamin D: high dose 1200 IU/d of vitamin D and low dose 400 IU/d of vitamin D. However, other studies in infantile period had only one experimental group—400 IU/d of vitamin D in comparison to placebo.

### Quality assessment

According to the Cochrane Collaboration’s tool for assessing risk of bias in randomized trials [16], the quality of trials was evaluated. The following criteria were considered (assessing at 3 levels such as low, high or unclear risk): random sequence generation, allocation concealment, blinding of participants and personnel, blinding of outcome assessment, incomplete outcome data, selective reporting and other bias.

### Statistical analysis

Statistical analysis of data was prepared in R (version 4.2.1). To evaluate the influence of vitamin D supplementation in experimental group compared to control, the relative risk (RR) with 95% confidence interval (CI) was calculated for dichotomous outcomes, while mean difference with 95% CI for continuous outcomes. Random effects model was used to calculate effect sizes. I^2^ statistics was used to evaluate the heterogeneity of studies: I^2^ < 40% may not be important; 30% < I^2^ < 60% means moderate heterogeneity; 50% < I^2^ < 90% means substantial heterogeneity; I^2^ > 75% means considerable heterogeneity [17]. To assess publication bias, funnel plot, Peters’ regression test (for dichotomous outcomes) and Egger's regression test (for continuous outcomes) were used. Results of this meta-analysis were considered statistically significant at *p* < 0.05.

## Results

### Search results

**Fig. 1:**
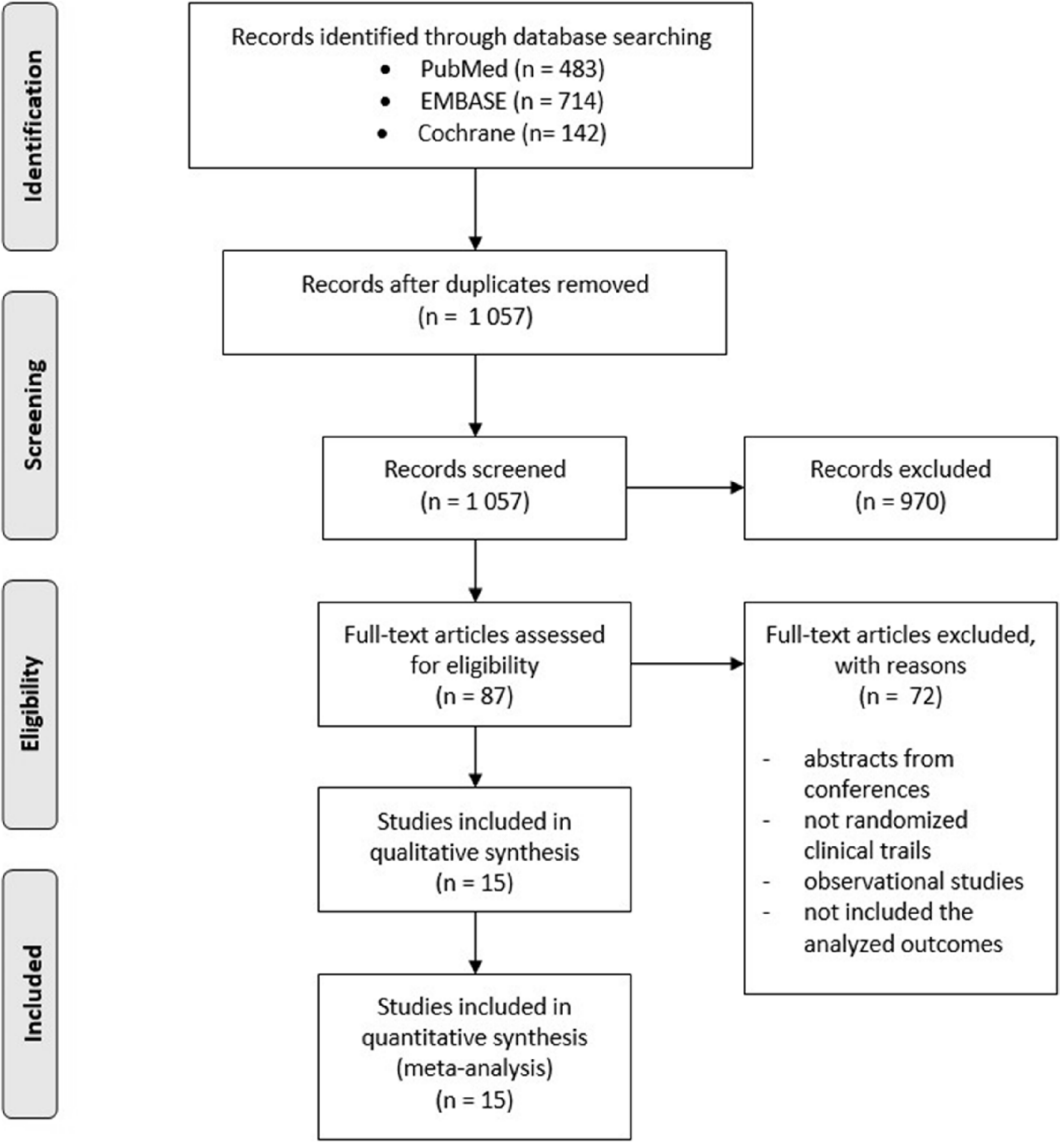
Study selection for meta-analysis

Literature search resulted in finding 1 057 articles after removal of duplicates (Fig. 1). During the first screening, we excluded 970 articles, such as meta-analyses, in vitro studies, studies on animals, case reports, observational studies and literature reviews. Moreover, we included articles written only in English. After full-text screening, 15 articles were qualified for the analysis.

All included studies are randomized controlled trials with control group concerning the vitamin D supplementation. Among these studies, three studies were carried out in pregnant women and contained the data from gestational period subjects [18–20], four studies contained data from infantile period subjects [15, 21–23], three from childhood period subjects [14, 24, 25], and five from adulthood period subjects [26–30]. The studies were carried out in different countries, such as Egypt [28], Spain [27], Denmark [19], Iran [26], U.S. [18, 20, 22], India [14, 24, 30], Ireland [25], United Kingdom [29], Finland [15], Australia [21, 23] and New Zealand [31]. Twelve of selected studies used placebo in control group [14, 18–27, 29], while two studies used standard asthma therapy in control group [28, 30]. Moreover, as study by Rosendahl et al. [15] included two experimental groups with varying doses of the vitamin D—1200 IU/d and 400 IU/d, therefore the lower dose was considered as a control group. Table 1 shows the characteristics of included studies.

**Table 1:** Characteristics of included RCTs

Studies	Type	Participants	Mean level of 25(OH)D at baseline [mean (SD)] or [median (IQR)]	Age	Mean age	Sex (girl/female)	Interventions	Treatment period	Observational period
**Gestational period**									
Litonjua et al., 2016 [18]	randomized, double-blind, placebo-controlled study	876 women with asthma, eczema or allergic rhinitis history (or in the biological father) 806 children	I: 23.3 (10.1) ng/mL C: 22.5 (10.1) ng/mL	18 – 39 years	I: 27.5 C: 27.3	I: 51% C: 45%	I: 4000 IU/d of vitamin D plus a prenatal vitamin containing 400 IU vitamin D C: placebo plus a prenatal vitamin containing 400 IU of vitamin D	pregnancy	asthma and wheezing were diagnosed in children up to 3 years of age
Chawes et al., 2016 [19]	randomized, double-blind, placebo-controlled study	581 healthy women	I: 31 (10) ng/mL C: 31 (10) ng/mL	*NA*	I: 32.5 C: 32.0	*NA*	I: 2400 IU/d plus a prenatal vitamin containing 400 IU of vitamin D C: placebo plus a prenatal vitamin containing 400 IU of vitamin D	pregnancy week 24 to 1 week postpartum	asthma and wheezing were diagnosed in children up to 3 years of age
Litonjua et al., 2020 [20]	a randomized, double-blind, placebo-controlled trial	876 women with asthma, eczema or allergic rhinitis history (or in the biological father) 806 children	I: 23.3 (10.3) ng/mL C: 22.6 (10.2) ng/mL	18 – 39 years	I: 27.5 C: 27.2	I: 50.5% C: 45.1%	I: 4000 IU/d of vitamin D plus a prenatal vitamin containing 400 IU of vitamin D C: placebo plus a prenatal vitamin containing 400 IU of vitamin D	pregnancy	asthma and wheezing were diagnosed in children up to 6 years of age
**Infantile period**									
Rosendahl et al., 2019 [15]	randomized, double blinded controlled trial	975 infants	Cord blood 30 µg: 81.3 (24) nmol/L 10 µg: 81.7 (28) nmol/L	from the age of 2 weeks	-	I: 50% C: 50%	30 µg: 1200 IU/d of vitamin D 10 µg: 400 IU/d of vitamin D	from 2 weeks to 24 months of age	asthma and wheezing were diagnosed up to 12 months of life
Rueter et al., 2020 [21]	randomized, double-blinded controlled trial	195 infants	Cord blood I: 67.8 (17.5) nmol/L C: 61.1 (14.2) nmol/L	before 28 days of age	I: 13.2 C: 12.8 days at randomization	I: 47.4% C: 45.9%	I: 400 IU/d of vitamin D3 C: placebo	for the first six months of life	asthma and wheezing were diagnosed up to 2.5 years of age
Hibbs et al., 2018 [22]	masked placebo-controlled randomized clinical trial	300 infants	I: 19.1 (15.7–28.0) ng/mL C: 21.0 (17.0–25.0) ng/mL	*NA*	I: 11 C: 13 days at randomization	I: 49.7% C: 39%	I: 400 IU/d of cholecalciferol C: placebo	until 6 months of age	asthma and wheezing were diagnosed up to 12 months of life
Rueter et al., 2019 [23]	a double-blind, placebo-controlled RCT	195 inflants	*NA*	before 28 days of age	I: 13.2 C: 12.8 days at randomization	I: 47.4% C: 45.9%	I: 400 IU/d of vitamin D3 C: placebo	for the first six months of life	wheezing was diagnosed up to 3 and 6 months of age
**Childhood period**									
Thakur et al., 2021 [24]	placebo‐controlled, blinded, randomized controlled trial	60 children with moderate persistent asthma	I: 15.8 (8.2) ng/mL C: 16.5 (9.9) ng/mL	6 – 11 years	I: 9 C: 8.7	I: 46.6% C: 40%	I: 2000 IU/d of vitamin D C: placebo + budesonide 400 μg and formoterol 24 μg daily	3 months	the C‐ACT score and FEV1 were measured at baseline and after 3 months
Jat et al., 2021 [14]	double-blind, randomized controlled trial	250 asthmatic children	I: 11.6 (4.6) ng/ml C: 10.8 (4.4) ng/ml	4 – 12 years	I: 8.2 C: 7.8	I: 71.2% C: 72.8%	I: 1000 IU/d of vitamin D C: placebo	9 months	the C‐ACT score and FEV1 were measured at baseline and after 9 months
Kerley et al., 2016 [25]	a double-blind, randomized, PL-controlled trial	44 children with asthma	I: 58 (39–69) (nmol/l) C: 51 (39–64) (nmol/l)	6–16 years	I: 10 C: 7	I: 35% C: 41%	I: 2000 IU/d of vitamin D_3_ C: placebo	15 weeks	the C‐ACT score and FEV1 were measured at baseline and after 15 weeks
**Adulthood period**									
Emami Ardestani et al., 2020 [26]	randomized, controlled clinical trial	132 mild-to-moderate asthma patients with vitamin D insufficiency and deficiency	Insufficient group I: 23.42 (2.64) ng/ml C: 23.64 (3.26) ng/ml Deficient group I: 11.29 (0.79) ng/ml C: 11.36 (0.75) ng/ml	above 18 years old	I: 42.77 C: 41.00	Insufficient group I: 33.3% C: 57.6% Deficient group I: 51.5% C: 39.4%	Insufficient group I: 1000 U/d of vitamin D C: placebo Deficient group I: 50,000 U/week of vitamin D to achieve serum 25(OH)D level > 20 ng/ml; followed by a maintenance dose of 1000 U/d C: placebo + asthma controller (Symbicort)	3 months	the ACT score and FEV1 were measured at baseline and after 3 months
Andújar-Espinosa et al., 2021 [27]	randomized, triple-blind, placebo-controlled, parallel-group study	112 adult asthmatic patients	I: 16.71 (6.71) ng/mL C: 17.48 (5.72) ng/mL	above 18 years old	I: 54.57 C: 56.61	I: 71.4% C: 83.9%	I: 16,000 IU/week of calcifediol C: placebo + usual asthma treatment	6 months	the ACT score and FEV1 were measured at baseline and after 6 months
Ali et al., 2017 [28]	an open-label prospective randomized controlled trial	82 patients with asthma	I: 18 (3.7–45) ng/mL C: 18.5 (3.5–54.7) ng/mL	18 to 65 years old	I: 43 C: 48	I: 62.8% C: 74.4%	I: 1 µg/d of alfacalcidol C: standard asthma treatment Vental inhaler as needed + Vental Compositum (mild asthma) + Foradil Aerolizer or Uniphyllin (moderate asthma) Higher dose of Beclomethasone or Prednisolone added (severe asthma)	4 months	FEV1 was measured at baseline and after 4 months
Martineau et al., 2015 [29]	a prospective randomized, placebo controlled, triple-blind study	250 adults with asthma	I: 49.8 (25.2) nmol/L C: 49.4 (24.2) nmol/L	16 to 80 years old	I: 49.4 C: 46.4	I: 56% C: 57%	I: 120 000 IU/2 months of vitamin D3 (Vigantol oil) C: placebo	12 months	the ACT score and FEV1 were measured at baseline and after 12 months
Nageswari et al., 2014 [30]	an open labeled, randomized comparative trial	68 patients with asthma	NA	35 to 65 years	I: 57.26 C: 56.23	I: 18% C: 16%	I: 1000 IU/d of vitamin D3 + Budesonide 400 μg + formoterol 24 μg C: Budesonide 400 μg + formoterol 24 μg	90 days	FEV1 was measured at baseline and after 90 days

*NA* not applicable, *IQR* interquartile range, *I* intervention group, *C* control group, *C‐ACT* childhood asthma control test, *ACT* asthma control test, *FEV1* forced expiratory volume in one second

### Quality assessment

The risk of bias assessment was conducted for 15 included RCTs. Eight of them are characterized by potential high risk of bias; while the other by low risk of bias. Additional file 1 shows the summary of the risk of bias assessment.

### Influence of vitamin D supplementation in gestational period

**Fig. 2:**
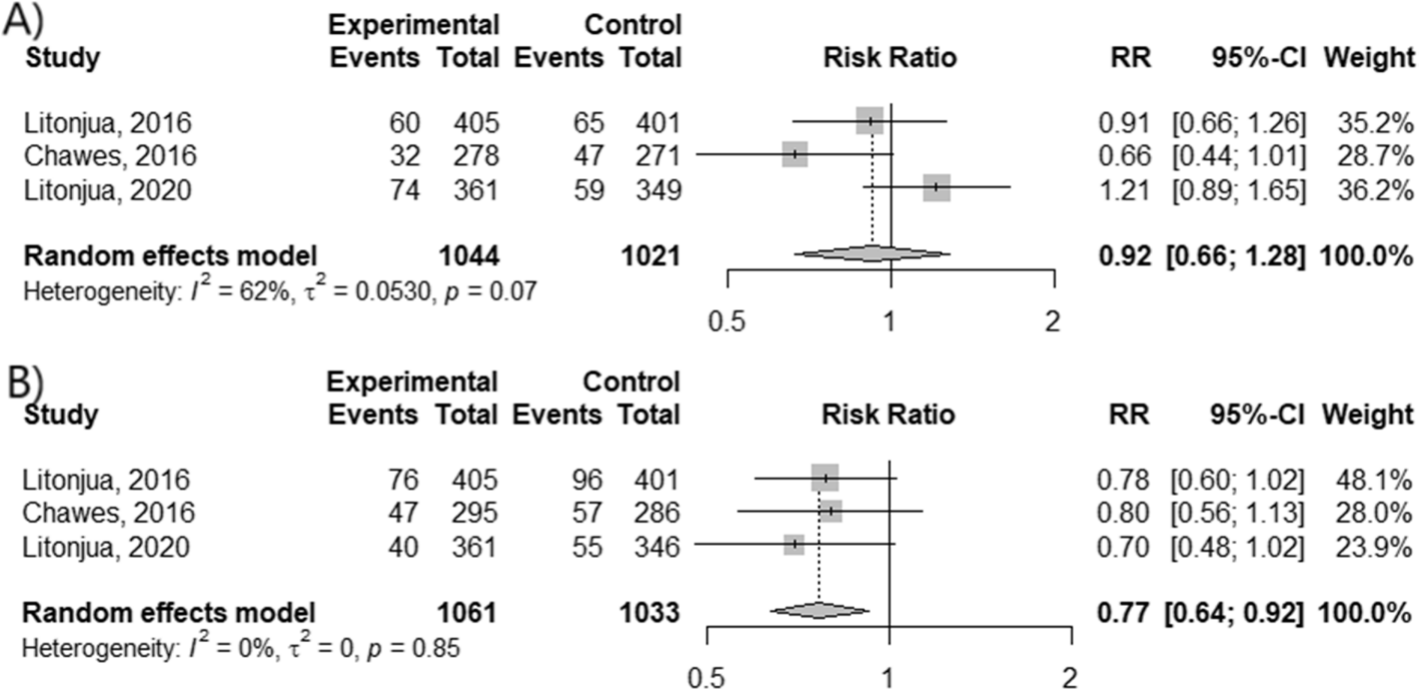
The efficacy of vitamin D supplementation during gestational period. The occurrence of (**A**) asthma diagnosis and (**B**) wheezing

We analyzed if the vitamin D supplementation by pregnant women can prevent the future asthma development in their children (Fig. 2 A, B). Data from clinical trials showed that vitamin D decreased the risk of wheezing incident by 23% (RR = 0.77; 95% CI [0.64; 0.92]; *p* < 0.0049, I^2^ = 0%). However, the decline in risk of asthma diagnosis rate was not statistically significant (*p* = 0.6361).

### Influence of vitamin D supplementation in infantile period

**Fig. 3:**
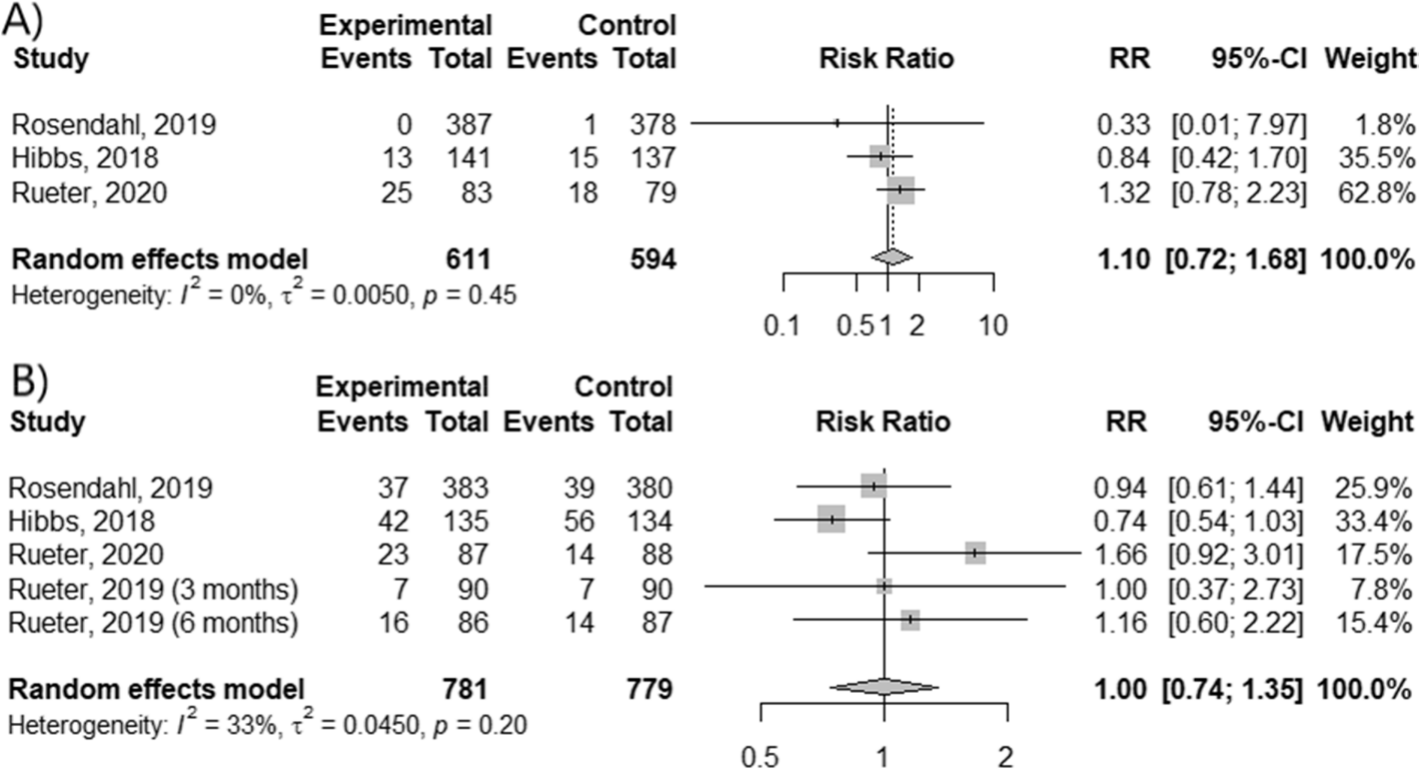
The efficacy of vitamin D supplementation during infantile period.  The occurrence of (**A**) asthma diagnosis and (**B**) wheezing

Unfortunately, meta-analysis of vitamin D supplementation in infant subjects showed no evident effect on asthma prevention (Fig. 3 A, B) as occurrence of asthma diagnosis and wheezing events was not significantly reduced by the intervention (*p* = 0.6659 and *p* = 0.9842, respectively). However, administration of high dose of vitamin D decreased the risk of asthma diagnosis in comparison to standard dose (RR = 0.33; 95% CI [0.01; 7.97]).

### Influence of vitamin D supplementation in childhood period

**Fig. 4:**
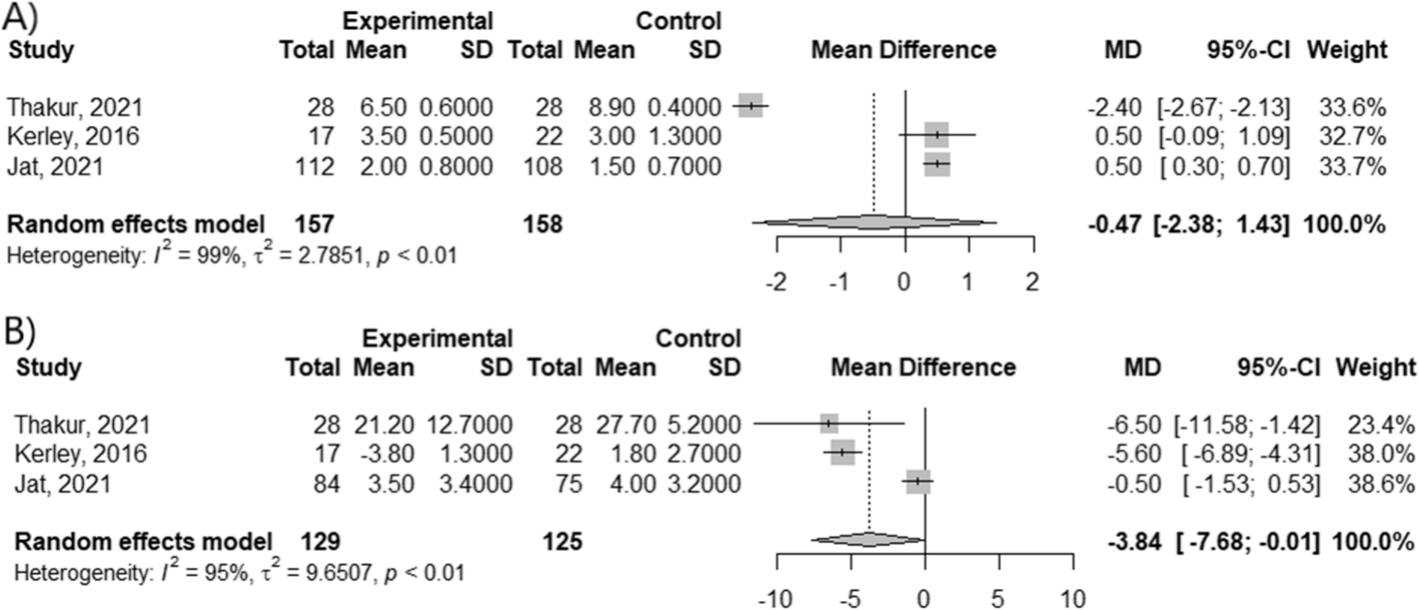
Mean difference in (**A**) change of C-ACT score and (**B**) change of FEV1

The meta-analysis of clinical trials from childhood period showed no difference between vitamin D supplementation and control groups in relation to change of the C-ACT score (MD = -0.47; 95% CI [-2.38; 1.42]; *p* = 0.6253; I^2^ = 99%) (Fig. 4A). However, apparent differences in FEV1 value (MD = -3.84; 95% CI [-7.68; -0.01]; *p* = 0.0497; I^2^ = 95%) (Fig. 4B) have been observed.

### Influence of vitamin D supplementation in adulthood period

**Fig. 5:**
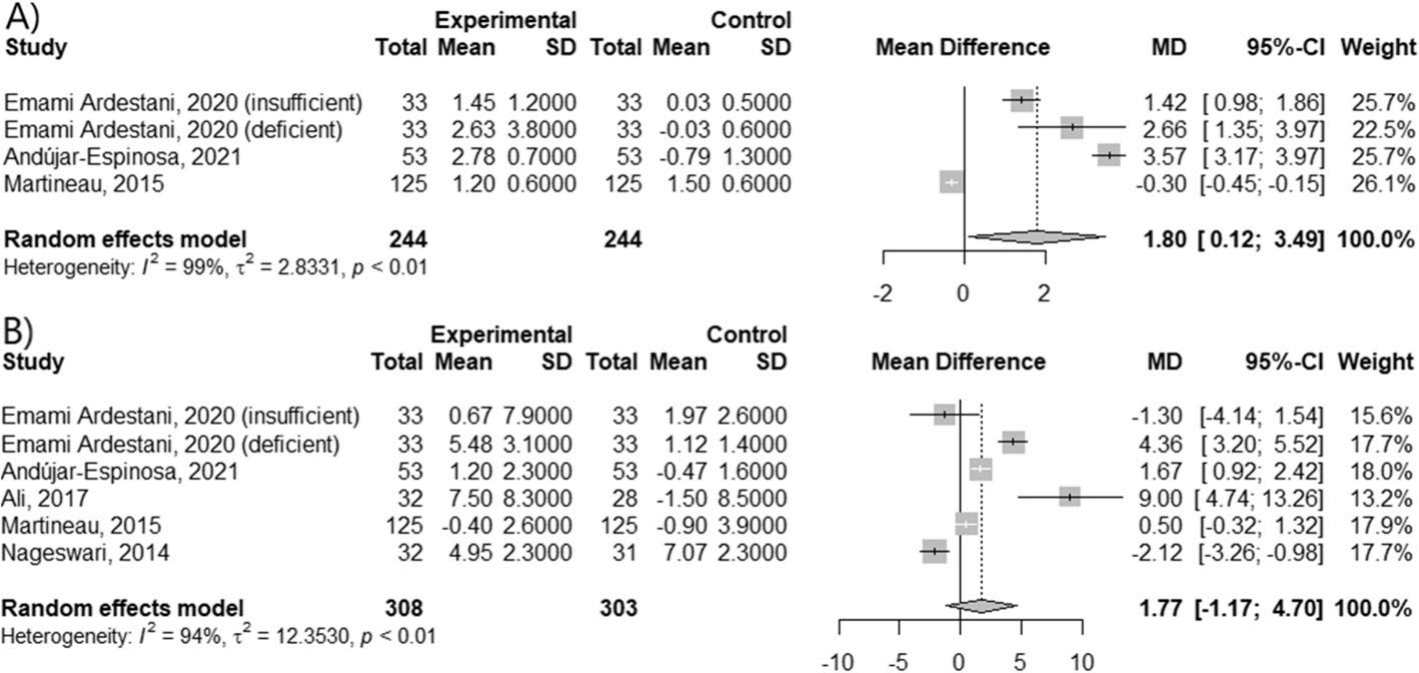
Mean difference in (**A**) change of ACT score and (**B**) change of FEV1

In the last stage of study, we checked the influence of vitamin D supplementation in adult patients (Fig. 5 A, B). There was a significant difference in ACT score change between vitamin D-supplied and control groups (MD = 1.80; 95% CI [0.12; 3.49]; *p* = 0.0359; I^2^ = 99%). Moreover, the beneficial effect was more noticeable in vitamin D-deficient patients (MD = 2.66; 95% CI [1.35; 3.97]), than in the vitamin D-insufficient patients (MD = 1.42; 95% CI [0.98; 1.86]). However, it had no impact on FEV1 value (MD = 1.77; 95% CI [-1.17; 4.70]; *p* = 0.2385; I^2^ = 94%).

### Publication bias

Additional file 2 shows the funnel plots for all investigated outcomes: the number of asthma and wheezing incidents occurring in gestational and infantile periods, the change of childhood asthma control test C‐ACT score and FEV1 in childhood period; as well as the change of asthma control test ACT score and FEV1 in adulthood period. Additionally, Peters’ regression test and Egger's regression test were performed to calculate publication bias for these outcomes. The results of Peters’ regression test and Egger's regression test showed that there was no evidence of publication bias for the association between vitamin D supplementation and the occurrence of asthma (*p* = 0.5660) and wheezing (*p* = 0.8898) in gestational period; the events of asthma (*p* = 0.1583) and wheezing (*p* = 0.4027) occurring in infantile period; the change of C‐ACT score (*p* = 0.9113) and FEV1 (*p* = 0.7138) in childhood period; and the change of ACT score (*p* = 0.2609) and FEV1 (*p* = 0.7531) in adulthood period, as *p* for outcomes was greater than 0.05.

## Discussion

Our meta-analysis of data gathered from 8 592 participants enrolled into 15 randomized clinical trials assessing influence of vitamin D supplementation in asthma prevention and treatment showed the varying results depending on participant’s life period. Vitamin D supplementation in pregnancy decreased the wheezing occurrence in children; whereas the supplementation during the infantile period had no apparent effect on given asthma parameters. Moreover, the vitamin D administration had negative effect on the FEV1 change before and after treatment in children, but had positive effect on the change of ACT score in adults.

It is clear that, Vitamin D supplementation during pregnancy affects asthma occurrence in children, but meta-analyses and studies show conflicting results. Multivariable logistic regression found the association between maternal level of 25(OH)D during the second trimester and wheeze or asthma events in 4–6 years old children [32]. A meta-analysis of 15 prospective studies enrolling total of 12 758 participants found a non-linear (U-shaped) relationship between maternal 25-hydroxy vitamin D levels and risk of childhood asthma [33]. Wei et al. [34] conducted a meta-analysis including a total number of 3666 mothers and children, which proved that there is no correlation between low vitamin D levels and an increased risk of asthma or wheezing. On the other hand, another study showed that vitamin D supplementation is important during pregnancy and can reduce the risk of asthma and wheezing occurrence in children [35]. Similar results were obtained in study by Parr et al. [36], in which they observed the positive relationship between vitamin D intake in pregnancy and lower asthma frequency. Meta-analysis by Pojsupap et al. [37] showed that high doses of vitamin D may prevent the disease exacerbations. Despite the fact, our study showed the wheezing-alleviating capabilities of vitamin D supplementation during pregnancy, while the supplementation during infantile period had no beneficial effect on asthma prevention. Similarly, prospective cohort study showed that risk of wheezing in early childhood was reduced by 35% after supplementation during pregnancy, but supplementation in early childhood did not prevent wheezing [38]. However, lower 25(OH)D level was detected in infants with recurrent wheezing [39].

In the childhood period, asthma is one of the most frequent chronic diseases [40]. A study conducted in Southern Jordan, demonstrated the correlation between 25(OH)D level in children and asthma severity symptoms [41]. Severe vitamin D deficiency was observed mainly in children with allergic rhinitis, asthma and wheezing [42]. Moreover, 25(OH)D level was positively correlated with FEV1 and FEV1/FVC in asthmatic children [43]. Supplementation of vitamin D in asthmatic children for 6 months, diminished the number of asthma exacerbations [44]. However, our meta-analysis showed no significant impact on FEV1 values before and after the treatment. Moreover, no change was noted in terms of C-ACT score. Similarly, a study by Thakur et al. [24] showed no difference in FEV1 value among analyzed groups. Other meta-analysis by Hao et al. [45] presents an increase in C-ACT score in vitamin D and control groups, as well as lack of impact on FEV1 and FVC% (forced vital capacity) values.

Inflammatory processes occurring in the respiratory tract are affected by vitamin D deficiency [10]. A meta-analysis of 27 clinical trials published between 2010 and 2018 confirms that asthma patients with low vitamin D levels also had lower FEV1 scores. Moreover, it has been proven that vitamin D positively affected lung function in children as well as in adults [46]. That was also confirmed in a meta-analysis of 14 randomized clinical trials. Vitamin D supplementation lowered the frequency of asthma exacerbations and had positive effect on the lung function in patients with vitamin D deficiency [47]. On the other hand, another meta-analysis by Manousaki et al. [48] showed no link between genetically-determined low 25(OH)D levels and an increased risk of asthma and other atopic diseases. In turn, 25(OH)D level was correlated with improved ACT score in patients with asthma, but lower level of 25(OH)D did not lead to asthma exacerbations. Moreover, 3 months-long supplementation in patients with low level of 25(OH)D increased ACT score in the uncontrolled asthma group, but did not significantly improve lung function in both the partly controlled and uncontrolled asthma groups [49], what is also in line with our results. However, a meta-analysis of 7 clinical trials with a total of 955 participants showed that the rate of asthma exacerbations requiring treatment with systemic glucocorticoids was reduced with vitamin D supplementation [50]. Moreover, in contrast to our results, case control study conducted by Babar et al. [51] showed the improvement of FEV1 after vitamin D supplementation. Large study that analyzed data from the U.S. National Health and Nutrition Examination Survey (NHANES) from 2001 to 2010 years showed the correlation between vitamin D insufficiency and episodes of asthma and wheezing as well as lower FEV1 [52]. We observed more significant improvement in FEV1 after vitamin D supplementation in deficient patients rather than in patients with vitamin D insufficiency in comparison to control group in study by Thakur et al. [26].

In summary, our meta-analysis shows that vitamin D intake can prevent asthma or wheezing as well as support asthma treatment, depending on the age of patients. However, our study has some limitations. Vitamin D supplementation efficacy can be affected by factors such as the study population, study region and sunlight exposure, asthma treatment and severity, leading to high heterogeneity in our results. However, we could not use subgroup analysis because of small number of analyzed studies. In turn, studies reported results using different measures and parameters, thus their recalculation to common measure may cause some discrepancies. The role of vitamin D in asthma is still controversial. Up to date published results are sparse, therefore only 15 studies were included in our meta-analysis. Nevertheless, our study shines a new light upon the role of vitamin D in chronic disease such as bronchial asthma in different periods of life.

## Conclusions

Vitamin D supplementation is still controversial and not fully researched topic in relation to bronchial asthma. In our meta-analysis, we showed that vitamin D supplementation has different effect on prevention and improvement of the asthma treatment in gestational, infantile, childhood and adulthood periods. That’s why, it is important to understand its mechanism of action as well as further investigate the role of vitamin D supplementation in asthma management during clinical studies.

## Supplementary Information

**Additional file 1: Figure S1.** Risk of bias of included studies.

**Additional file 2: Figure S2.** Funnel plots for the associations of between vitamin D supplementation and asthma.

## Data Availability

All data generated or analyzed during this study are included in this published article and its additional files.

## Abbreviations

FEV1: Forced expiratory volume in one second
FVC%: Forced vital capacity
RCTs: Randomized clinical trials
RR: The relative risk
CI: Confidence interval
C‐ACT: Childhood asthma control test
ACT: Asthma control test
MD: Mean difference
