# Supplementary material for: Relationship between vitamin D and asthma from gestational to adulthood period: a meta-analysis of randomized clinical trials
Source: BMC Pulm Med. 2023 Jun 17;23:212. doi: 10.1186/s12890-023-02514-4 (PMC10276459; doi:10.1186/s12890-023-02514-4)
Supplement: Supplementary file 1 — Additional file 1: Figure S1. Risk of bias of included studies. [file 12890_2023_2514_MOESM1_ESM.docx]

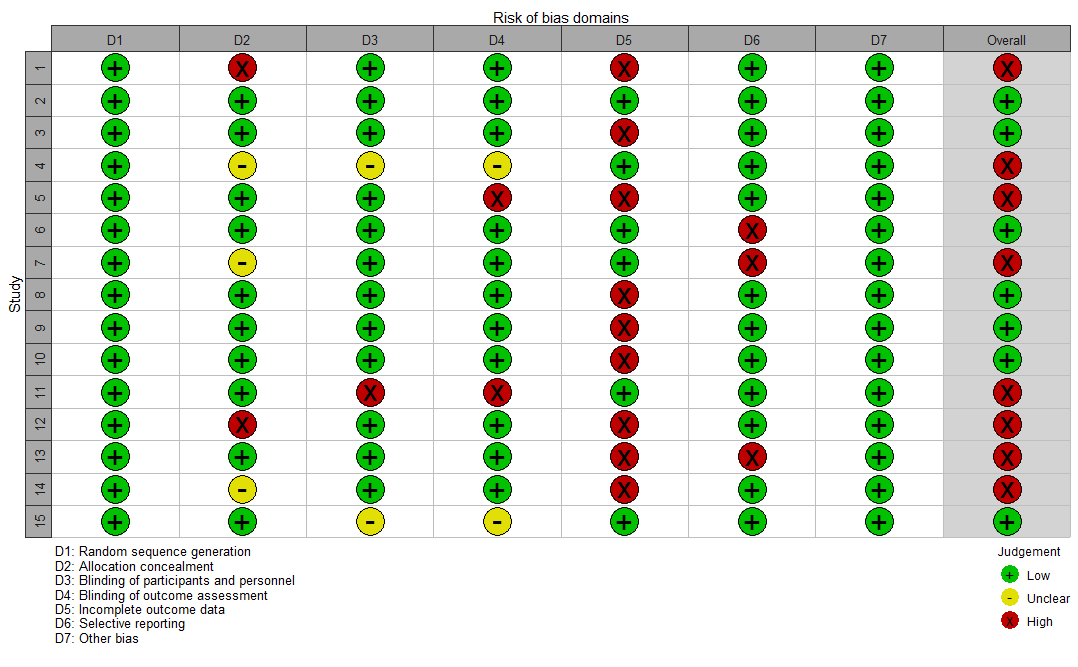
**Figure S1.** Risk of bias of included studies.

(1) Ali et al, 2017; (2) Andújar-Espinosa et al, 2021; (3) Chawes et al, 2016; (4) Emami Ardestani et al, 2020; (5) Hibbs et al, 2018; (6) Jat et al, 2021; (7) Kerley et al, 2016; (8) Litonjua et al, 2016; (9) Litonjua et al, 2020; (10) Martineau et al, 2015; (11) Nageswari et al, 2014; (12) Rosendahl et al, 2019; (13) Rosendahl et al, 2019; (14) Rosendahl et al, 2020; (15) Thakur et al, 2021.
