# Supplementary figures and images for: Relationship between vitamin D and asthma from gestational to adulthood period: a meta-analysis of randomized clinical trials
Source: BMC Pulm Med. 2023 Jun 17;23:212. doi: 10.1186/s12890-023-02514-4 (PMC10276459; doi:10.1186/s12890-023-02514-4)

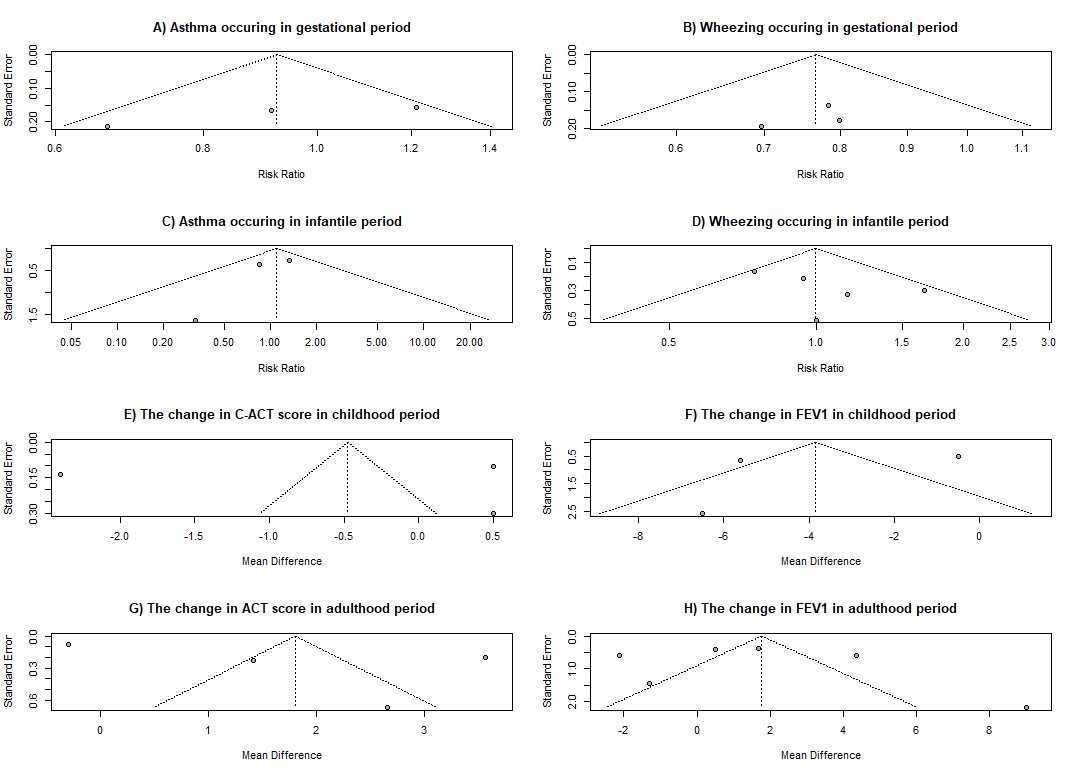


**Figure S2**. Funnel plots for the associations of between vitamin D supplementation and asthma.

Supplement: Supplementary file 2 — Additional file 2: Figure S2. Funnel plots for the associations of between vitamin D supplementation and asthma. [file 12890_2023_2514_MOESM2_ESM.docx]
